# Cancer risk perceptions and distress among women attending a familial ovarian cancer clinic

**DOI:** 10.1054/bjoc.2000.1651

**Published:** 2001-03

**Authors:** A Cull, A Fry, R Rush, C M Steel

**Affiliations:** Psychology Research Group, Imperial Cancer Research Fund (ICRF) Medical Oncology Unit, Western General Hospital, Edinburgh, EH4 2XU; School of Biological and Medical Sciences, University of St Andrews, Bute Medical Building, St Andrews, Fife, KY16 9TS, UK

**Keywords:** ovarian cancer, psychological distress, cancer risk, genetic counselling

## Abstract

Of 230 women referred to a familial ovarian cancer clinic, 196 (85%) completed a questionnaire before they attended. The data collected included pre-counselling risk perceptions and an assessment of distress. Respondents were more likely to underestimate (44%) than overestimate (19%) their risk. Those with a family history of breast and ovarian cancer (HBOC) were particularly likely to underestimate their ovarian cancer risk. The variables assessed in this study – sociodemographic, family history, distress, anxiety proneness, coping style and beliefs about health control – explained little of the observed variation in accuracy of risk perception. On the General Health Questionnaire (GHQ-30) 30% of the sample obtained scores above the cut-off (≥ 6) recommended for screening for ‘case-level’ psychological distress. Women exhibiting case-level distress were more likely to overestimate their risk (OR = 2.3). On univariate analysis low internal locus of control was associated with ‘case-level’ distress (*P* = 0.008). On multiple regression the best predictors of ‘caseness’ were high-trait anxiety, being a graduate and inaccurate risk perception. There was no difference in the level of distress shown by women with HBOC vs. those with a history of ovarian cancer only. Implications of these findings for the counselling needs of the women are discussed. The effectiveness of the clinic in improving the accuracy of risk perceptions and relieving distress is being assessed. © 2001 Cancer Research Campaign http://www.bjcancer.com


*British Journal of Cancer* (2001) **84**(5), 594–599
© 2001 Cancer Research Campaign
doi: 10.1054/ bjoc.2000.1651, available online at http://www.idealibrary.com on IDEAL

### Cancer risk perceptions and distress among women attending a familial ovarian cancer clinic

**A Cull[1], A Fry[1], R Rush[1] and CM Steel[2]**

[1]Psychology Research Group, Imperial Cancer Research Fund (ICRF) Medical Oncology Unit, Western General Hospital, Edinburgh EH4 2XU; [2]School of Biological and Medical Sciences, University of St Andrews, Bute Medical Building, St Andrews, Fife KY16 9TS, UK

**Summary** Of 230 women referred to a familial ovarian cancer clinic, 196 (85%) completed a questionnaire before they attended. The data collected included pre-counselling risk perceptions and an assessment of distress. Respondents were more likely to underestimate (44%) than overestimate (19%) their risk. Those with a family history of breast and ovarian cancer (HBOC) were particularly likely to underestimate their ovarian cancer risk. The variables assessed in this study – sociodemographic, family history, distress, anxiety proneness, coping style and beliefs about health control – explained little of the observed variation in accuracy of risk perception. On the General Health Questionnaire (GHQ-30) 30% of the sample obtained scores above the cut-off (≥ 6) recommended for screening for 'case-level' psychological distress. Women exhibiting case-level distress were more likely to overestimate their risk (OR = 2.3). On univariate analysis low internal locus of control was associated with 'case-level' distress ($P = 0.008$). On multiple regression the best predictors of 'caseness' were high-trait anxiety, being a graduate and inaccurate risk perception. There was no difference in the level of distress shown by women with HBOC vs. those with a history of ovarian cancer only. Implications of these findings for the counselling needs of the women are discussed. The effectiveness of the clinic in improving the accuracy of risk perceptions and relieving distress is being assessed. © 2001 Cancer Research Campaign http://www.bjcancer.com

**Keywords**: ovarian cancer; psychological distress; cancer risk; genetic counselling

A growing number of clinics has been set up to offer genetic counselling to individuals whose family history of cancer suggests inherited susceptibility which puts them at increased risk of developing cancer, often at a relatively early age. The services offered by these clinics need to be adequately evaluated with respect to both medical and psychosocial outcomes to inform future practice. The establishment of a specialist ovarian cancer family clinic in SE Scotland (Mackay et al, 1995) provided a unique opportunity to assess the psychological impact on the women attending. We had begun assessing the knowledge, attitudes, emotional and behavioural responses of women attending a familial breast cancer clinic (Cull et al, 1999). We therefore sought to collect comparable data in this setting.

The lifetime risk of developing ovarian cancer for women in the general population in Scotland is 1 in 75 (1.3%). For women with one affected relative the risk is of the order of 3–4% and may be as high as 40% for a woman with two or more affected relatives (Jacobs and Lancaster, 1996). The highly penetrant *BRCA1* gene is thought to account for 5% of ovarian cancers among women under the age of 70 (Stratton et al, 1997). This proportion may be higher among young women (Ford et al, 1995). Other less penetrant genes are also thought to be implicated but there is less evidence available about the proportion of cases which can be attributed to these genes. Although in some families the inherited predisposition appears to be specific to ovarian cancer, the most common clinical pattern is in association with breast cancer.

There is a lack of evidence about how best to manage women with a family history of ovarian cancer. In contrast to screening for breast cancer, no screening method for ovarian cancer has yet been shown to be effective in early detection or in reducing mortality. Prophylactic oophorectomy, with its attendant side-effects and unproven efficacy, is probably the most effective means of reducing the risk of death from ovarian cancer currently available to women at increased risk. It is not clear whether the risk of psychological morbidity is greater for women at increased risk of developing cancer at more than one body site.

When the present study was planned there were scant published data about the psychological responses of women with ovarian cancer risk. The available data need to be interpreted with attention to how the samples were derived. In the US high levels of psychological distress among first degree relatives (FDRs) of ovarian cancer patients were associated with their own increased risk as well as in reaction to their relative's illness (Daly and Lerman, 1993). In the UK women who volunteered early for a familial ovarian cancer register were well informed about ovarian cancer, uncertain about genetic issues but not particularly anxious (Green et al, 1993). Wardle (1995) assessed two groups of respondents (with/without a FDR with ovarian cancer) to a national (UK) advertisement for participants for a study of ovarian cancer screening. Both these self-selected groups had higher perceptions of their personal risk of ovarian cancer and higher levels of cancer worry than general population controls. In Canada, among women assessed after attending a familial ovarian cancer clinic, half correctly reported their risk (as high/moderate/low), 26% overestimated and 17% minimized their risk (Robinson et al, 1997). The minimizers were significantly less distressed than the accurate or over-estimators, of whom 40% were significantly depressed and 20% were highly anxious.

A variety of personal characteristics have been implicated in mediating the relationship between perceived threat to health, distress and health protective behaviours. A tendency to seek out information about threat – 'monitoring' (Miller, 1987) – was associated with a

*Received 4 April 2000*
*Revised 5 December 2000*
*Accepted 5 December 2000*

*Correspondence to:* A Cull

**Table 1** Criteria for referral to familial ovarian cancer clinic *at the time of this study* (Mackay et al, 1995)

| **Any woman who has :** |
|---|
| • ≥ 1 first degree relative with epithelial ovarian cancer under 55 years of age |
| • ≥ 1 first degree relative with *both* breast *and* epithelial ovarian cancer at any age |
| • 1 first degree relative with epithelial ovarian cancer at any age *and* ≥ 1 additional first or second degree relative with breast or ovarian cancer |
| • an actual or perceived family history of cancer causing undue anxiety |

In this context a first degree relative is a mother, sister or daughter and a second degree relative is grandmother, aunt or first cousin.

higher perceived risk of ovarian cancer, more intrusive thoughts about cancer and higher levels of distress in women with a FDR with ovarian cancer (Schwartz et al, 1995) and with a higher level of cancer worry among screening attenders (Wardle, 1995). Beliefs about the controllability of one's health in the face of inherited susceptibility to cancer may also influence psychological adjustment. Among women with an FDR with breast and/or ovarian cancer, those with high-risk perceptions and low levels of perceived control were most vulnerable to distress (Audrain et al, 1997).

Our ovarian cancer family clinic is multidisciplinary and based in a 'Well Woman Clinic' in the community (Mackay et al, 1995). Patients were typically referred by their general practitioner according to criteria based on their family history (Table 1). A significant proportion were referred from the familial breast cancer clinic. This study was designed to address the following questions:

1. What were the characteristics of women who presented at the clinic? In particular did they overestimate their risk and were they highly distressed?
2. Did women with a family history of breast *and* ovarian cancer (HBOC) perceive their risk as greater and were they more distressed than women with a history of ovarian cancer (HOC) only?
3. Can we predict women's presenting risk perceptions and levels of distress from their sociodemographic details, family history or psychological characteristics?

### SAMPLE

A consecutive series of 230 women, with a family history of ovarian cancer, newly referred for counselling about their risk of developing ovarian cancer were eligible to take part in the study. Data were collected between June 1994 and December 1998.

### MEASURES

### Sociodemographic and family history characteristics

The following data were recorded: age; marital status (married/ living with partner vs not); children (yes/no); daughters (yes/no); educational attainment (university level/less than university); family history (history of ovarian cancer – HOC vs. history of breast *and* ovarian cancer – HBOC).

### Risk estimate

Women were asked to select from 10 categories the response (e.g. inevitable, 1 in 2... <1 in 200, very unlikely) which they believed to be a) the risk for a woman in the general population and b) their own lifetime risk of developing ovarian cancer (adapted from Evans et al, 1993, 1994). They were also asked to rate their personal susceptibility to developing ovarian cancer: not very/ moderately/very susceptible.

### Psychological distress

The General Health Questionnaire (GHQ-30) with a cut-off score of ≥ 6, was used to screen for clinically significant levels of psychological distress and dysfunction. Published data from the general population are available for comparison (Goldberg and Williams, 1988).

### Psychological characteristics

#### *Anxiety proneness*

The Spielberger State-Trait Anxiety Inventory (STAI) (Spielberger, 1983) was used to measure anxiety proneness (trait anxiety). Knight et al (1983) collected STAI data from a general population sample in an area of New Zealand with a strong history of immigration from Scotland. The STAI trait anxiety scores which they present by age (in 10-year bands) and sex offer more appropriate reference data for this study than those in the STAI manual which are derived from employees in the US Federal Aviation Administration.

#### *Beliefs about control of health*

The Health-related Locus of Control Scale (Wallston and Wallston, 1978) was used to assess the extent to which the women attributed their health to internal (i.e. own behaviour), external (e.g. doctors) or chance factors. The 9 items with the highest item-subscale correlations were selected (Marks et al, 1986). This short form allowed the role of locus of control to be explored while keeping the burden on respondents to a minimum.

#### *Coping style*

The Miller Behavioural Style Scale (Miller, 1987) was designed to assess the propensity of people to seek out ('monitor') or avoid ('blunt') information about threatening events. The short form presents 2 scenarios (Steptoe, 1989) to which respondents select their most likely reaction from a fixed choice of 'monitoring' and 'blunting' responses.

### PROCEDURE

Referred women were sent a family information sheet to complete and return by post. The family history given was verified and extended by reference to other sources e.g. cancer registry, hospital records etc. The woman's risk of ovarian cancer was estimated before a clinic appointment was offered. The assessment package for this study was posted to women with their clinic appointment and returned when they attended the clinic. A geneticist counselled women about their family history. Women at low risk were discharged from follow-up. Women at increased risk (> 3% lifetime risk) were seen by a gynaecologist and offered surveillance by clinical examination, Ca 125 testing and pelvic ultrasound (by separate appointment) to screen for ovarian cancer. At the time of this study genetic testing was not available to the women attending this clinic. For women at high risk (>5% lifetime risk), the geneticist rehearsed the issues in genetic testing

© 2001 Cancer Research Campaign 

and the gynaecologist discussed prophylactic surgery as a risk management option. For the purposes of this study a consultant geneticist reviewed all the casenotes. The women were categorized as being at high, moderate or low risk on the basis of their age and family history.

### STATISTICAL ANALYSIS

Descriptive statistics were generated to describe the study population. The associations between explanatory variables and ordered groups of risk estimates (very/moderately/not very susceptible; high/moderate/low risk) were examined using the non-parametric trend test (Cuzick, 1985). The chi-square test for trend was used to compare proportions across these ordered groups. The Mann-Whitney test was used to compare personal risk estimates for the 2 sub-groups (HOC vs. HBOC). Comparisons between two independent samples were made using two-sample *t*-tests. Univariate analyses were undertaken to explore relationships of accuracy of personal risk perception and distress with each other and with sociodemographic, family history and personal characteristics. The results informed the forward stepwise selection of variables entered into the logistic regression analyses undertaken to construct predictive models of under- and overestimating risk and for 'case-level' distress. The criterion for entering variables into the model was $P \leq 0.05$ and for removing them $P \geq 0.1$. The data were analysed using the statistical package SPSS for Windows (1999).

### RESULTS

230 women were eligible for inclusion in the study. 196 of them (85%) completed baseline assessments and returned them when they attended the clinic. 15 women attended the clinic but failed to return their baseline assessment and 14 women neither attended the clinic nor completed baseline assessments. As a result of administrative failures 5 women were not contacted.

### Sociodemographic and family history characteristics

Participants in this study ranged in age from 21.4 to 69.6 years (mean = 42.1 years, SD = 9.8, *n* = 196). The majority (74%) were married or cohabiting. Of the 75% who had children, 75% had 1 or more daughters. 40% had received secondary education to age 16; 15% to age 18; 17% had had some tertiary education and 28% were university graduates. 85 women (44%) also had a family history of breast cancer.

### Risk assessment

#### *Risk estimate – ratios*

184 women each endorsed one of the ratios offered to indicate their estimate of the risk for a woman in the general population of developing ovarian cancer. 36% were in the correct range i.e. 1 in 50–1 in 100; 28% endorsed values ≤ 1/200; 29% were in the range 1/20–1/10; 7% endorsed values ≥ 1/4. Of 185 women returning personal risk estimates: 5 (3%) believed it inevitable they would develop ovarian cancer and 20 (11%) set their risk at ≤1/200. The remaining personal risk estimates showed a bimodal distribution between these extremes (Figure 1). Estimates of both general population and personal risk were available from 184 women: 78% set their own risk at least twice the general population risk whatever they believed that to be and 92 (50%) set their risk at ≥ 3 times their estimate for the general population. Surprisingly, 8% (*n* = 15) set their risk *lower* than the risk they endorsed for the general population by a factor of ≤0.5.

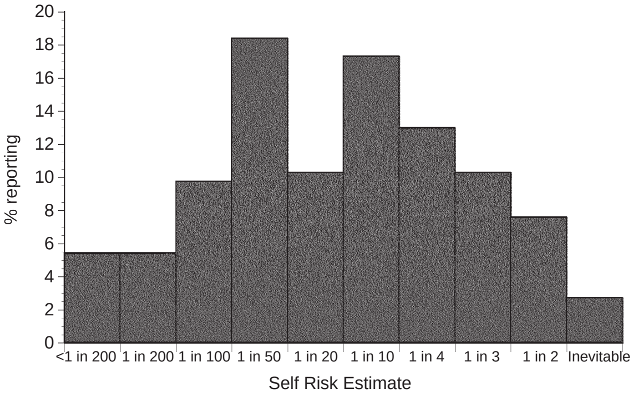

**Figure 1** Distribution of estimates of personal risk of developing ovarian cancer among women attending ovarian cancer family clinic (N= 185)

**Table 2** The relationship between women's estimates of personal risk/susceptibility to ovarian cancer and risk category assigned by geneticist

| | Personal risk estimate – ratio endorsed | Personal susceptibility very | mod | not very | |
|---|---|---|---|---|---|
| Risk category assigned: | | | | | |
| high | Range: inevitable – ≤1 in 200<br>Median= 1 in 10<br>(n=97) | 23<br>(24%) | 61<br>(62%) | 14<br>(14%) | N=98 |
| moderate | Range: inevitable – ≤1 in 200<br>Median = 1in 10<br>(n=55) | 9<br>(15%) | 41<br>(71%) | 8<br>(14%) | N=58 |
| low | Range: 1in2 – ≤1 in 200<br>Median = 1 in 50<br>(n=32) | 2<br>(6%) | 25<br>(76%) | 6<br>(18%) | N=33 |

#### *Personal susceptibility*

Prior to attending the clinic 67% (*n* = 128/190) rated themselves as moderately susceptible to developing ovarian cancer; 34 women (18%) rated themselves very susceptible and the remaining 28 (15%) rated themselves not very susceptible. The use of these verbal descriptors was significantly related to the numerical risks endorsed (z = 5.32, $P < 0.0001$). The range of ratios endorsed showed considerable overlap but women who rated themselves 'very susceptible' did endorse higher personal risk ratios (median = 1/4, range: inevitable – 1/200) than 'moderately' (median = 1/20, range: 1/2 – ≤1/200) or 'not very' susceptible women (median = 1/50, range: 1/3 – ≤1/200).

#### *Comparison of personal and professional risk assessments*

Data were available from the notes of 195 women of whom 102 (52%) were deemed to be at high risk. For 59 (30%) the risk assigned was 'moderate' and for the remaining 34 women (17%) the risk was assessed as low. The relationships between risk categories assigned by the geneticist and the women's pre-clinic assessments of their own numerical risk and susceptibility are shown in Table 2.

Women whose risk of developing ovarian cancer was judged by the geneticist to be low had themselves returned significantly lower numerical estimates of their risk than women judged to be at moderate or high risk (z = 2.05, $P = 0.04$). There was no

© 2001 Cancer Research Campaign

**Table 3** Psychological Characteristics by Accuracy of Risk Estimate

| | Underestimators | | | Overestimators | | |
|---|---|---|---|---|---|---|
| | N | Mean | SD | N | Mean | SD |
| Trait Anxiety | 75 | 39.0 | 9.2 | 36 | 41.1 | 9.2 |
| Locus of Control: | | | | | | |
| Chance | 80 | 8.0 | 3.2 | 35 | 8.9 | 3.5 |
| Internal | 80 | 13.5 | 2.7 | 35 | 13.1 | 2.6 |
| External | 80 | 6.9 | 3.6 | 35 | 7.4 | 3.4 |
| Coping Style: | | | | | | |
| Monitoring | 79 | 3.8 | 1.6 | 36 | 3.9 | 1.8 |
| Blunting | 79 | 2.1 | 1.2 | 36 | 1.8 | 0.9 |

significant association between the risk category assigned by the geneticist and the women's own ratings of their susceptibility ($\chi^2$ = 5.58, df = 4, $P$ = 0.23). For 37% of the sample (70/189 women) the risk category to which they were assigned accorded with their rating of their susceptibility – 'accurate estimators'. For 83 women (44%) – 'underestimators' – the risk assigned was higher and for 36 of them (19%) – 'overestimators' – lower than their own prior rating of their susceptibility.

### *Women with HBOC compared with women with HOC*

Prior to attending the clinic there were no significant differences in personal risk estimates (Mann-Whitney U = 4024.0, $P$ = 0.66) nor in personal susceptibility ratings ($\chi^2$ = 2.58, df = 2, $P$ = 0.28) between these two groups of women. As expected the HBOC women were assigned a higher risk than the HOC women ($\chi^2$ = 15.6, df = 2, $P$ < 0.0005). This implies then that the HBOC women were more likely to underestimate their risk. Among the women who underestimated their risk at baseline, 58% (48 women) had a history of breast and ovarian cancer while among the overestimators the proportion was only 28% (10 women). Among 30 HBOC women who had been referred from the familial breast cancer clinic 73% ($n$ = 22) underestimated their risk of ovarian cancer.

### Psychological distress

### *GHQ*

The mean GHQ score of women attending this clinic was 4.5 (SD = 6.4, $n$ = 194). 59 women (30%) scored above the cut-off (5/6) for screening for 'case-level' distress. Women who overestimated their susceptibility to ovarian cancer had a significantly higher mean GHQ score (mean = 6.7, sd = 8.3, $n$ = 34) than 'underestimators' (mean = 3.5, SD = 5.4, $n$ = 83; t = 2.46, df = 115, $P$ = 0.02) and a higher proportion of 'cases' (47% vs. 23%, respectively).

### *Women with HBOC compared with women with HOC*

There were no significant differences in mean GHQ scores between these sub-groups of women. The proportion of 'cases' was somewhat lower among HBOC women (26% vs. 34%) but this difference was not statistically significant.

### Psychological characteristics

For the sample as a whole ($n$ = 187) the mean trait anxiety score was 40.1 (SD = 9.0). Mean scores from 192 women were calculated for the health-related locus of control and coping style scales: self (internal) mean = 13.2 (SD = 2.8); others (external) mean = 7.2 (SD = 3.4); chance mean = 8.2 (SD = 3.2); monitoring mean = 3.7 (SD = 1.7) and blunting mean = 1.9 (SD = 1.2).

### *Women with HBOC compared with women with HOC*

There were no significant differences in scores on any of the measures used.

### Predicting accuracy of initial risk perception

We first conducted univariate analyses to explore the relationships between overestimating the risk (vs. not) then, separately, underestimating (vs. not), and the sociodemographic, family history, distress and the psychological variables. Overestimators were significantly more likely to have HOC than HBOC ($\chi^2$ = 4.5, df = 1, $P$ = 0.03) and to exhibit case-level distress on GHQ ($\chi^2$ = 5.0, df = 1, $P$ = 0.03). Underestimators were conversely significantly more likely to have HBOC ($\chi^2$ = 13.2, df = 1, $P$ < 0.0005) and less likely to be GHQ 'cases' ($\chi^2$ = 4.6, df = 1, $P$ = 0.03). None of the other relationships was significant. The mean scores on the psychological measures are given for under- and overestimators separately in Table 3.

Separate multivariate logistic regression analyses were conducted to identify independent predictors of over- and under-estimators. The variables considered in these models were those found to be associated with over-/under-estimating at the 5% significance level on univariate analyses i.e. GHQ 'caseness' and HBOC vs HOC. A forward stepwise selection procedure was used. One variable was significant ($P$ < 0.05) in each model: women exhibiting 'case-level' distress were more likely to overestimate their risk (OR = 2.33, CI: 1.09–4.99); HBOC women were more likely to underestimate their risk (OR = 0.34, CI: 0.19–0.63).

### Predicting 'case-level' distress at first presentation

Univariate analyses were conducted to determine the factors associated with 'case-level' GHQ scores. The variables considered were as for risk perception above. Of the sociodemographic variables only education was significantly related to distress. The proportion of women exhibiting 'case-level' distress was significantly higher among university graduates than among the less well educated ($\chi^2$ = 10.4, df = 1, $P$ = 0.001). Neither family history nor the women's ratings of their susceptibility to ovarian cancer were related to 'caseness' but accuracy of risk perception was significant ($\chi^2$ = 6.8, df = 2, $P$ = 0.03). Of the psychological variables trait anxiety, chance and internal locus of control were all significantly related to 'caseness'. Mean scores for the psychological variables for 'cases' and 'not cases' are given in Table 4.

Women who scored as GHQ 'cases' were more anxiety prone (t = 6.73, df = 183, $P$ < 0.0005), more likely to ascribe control over their health to chance (t = 1.96, df = 189, $P$ = 0.05) and less likely to feel that their health was under their own control (t = 2.67, df = 189, $P$ = 0.008).

Multivariate logistic regression was conducted using the variables found to be significant ($P$ < 0.05) on univariate analysis with a forward stepwise procedure (Table 5). The category 'accurate estimator' was used as the reference against which over- and under-estimators were compared. University educated women and overestimators are significantly more likely to exhibit 'case-level' scores on the GHQ. The model predicts 39% of the variation in 'caseness'.

### DISCUSSION

Ultimately familial cancer clinics aim to reduce cancer mortality and morbidity. They seek to achieve this by identifying and counselling

© 2001 Cancer Research Campaign 

**Table 4** Psychological Characteristics by 'Case-Level' Distress (GHQ30 Score >5)

| | 'Case' | | | 'Non-Case' | | |
|---|---|---|---|---|---|---|
| | N | Mean | SD | N | Mean | SD |
| Trait Anxiety | 59 | 45.9 | 9.5 | 126 | 37.3 | 7.4 |
| Locus of Control: | | | | | | |
| Chance | 59 | 8.8 | 3.2 | 132 | 7.9 | 3.2 |
| Internal | 59 | 12.4 | 2.8 | 132 | 13.5 | 2.7 |
| External | 59 | 7.6 | 3.2 | 132 | 7.0 | 3.5 |
| Coping Style: | | | | | | |
| Monitoring | 59 | 4.0 | 1.8 | 131 | 3.6 | 1.6 |
| Blunting | 59 | 1.9 | 1.3 | 131 | 1.9 | 1.1 |

asymptomatic, at-risk individuals about cancer prevention and early detection. They may also have a role in educating and reassuring those whose risk is not sufficiently elevated to warrant specialist surveillance. To be cost-effective these clinics need predominantly to attract people who are at increased risk of cancer. They also need to be able to give information, with all its attendant uncertainty, in such a way that people can use it to make informed health care choices and without causing adverse psychological consequences.

This study describes the characteristics of women who attended a specialist familial ovarian cancer clinic. Referral criteria for this clinic had been circulated to GPs and relevant clinics (Mackay et al, 1995). Hence the majority of women in this study had been referred by a doctor and were at at least moderately increased risk of developing ovarian cancer. Data were not available from the 6% of referrals who failed to attend. These cannot be regarded as missing at random. Our hypothesis is that these women were anxious about their cancer risk and coping by avoidance ('blunting'). Compliance with the baseline assessment was excellent (93%) among 'attenders.' However the missing data probably also represent a defensive response from at least a proportion of the non-compliant women. Our data need to be interpreted in the light of this potential bias. It should be noted that referral criteria for this clinic are now more strict, to conform with those of the UKCCCR National Familial Ovarian Cancer Screening Study (Jacobs et al, 1997).

In common with observations from other health protection programmes (Audrain et al, 1995) there was an over-representation of well-educated women in our sample. 44% were at increased risk of both breast and ovarian cancer. 30 (35%) of the HBOC women had been referred from the breast cancer family clinic. Only 12 of them had been included in our study of that clinic (Cull et al, 1999). This was not considered a sufficiently large proportion of that sample ($n$ = 486) to invalidate comparison between that study and this one.

We were aware of the lack of consensus among professionals about how best to communicate about risk and the reservations of Hallowell and Richards (1997) about the meaning of numerical risk information to the women concerned. We therefore investigated the women's use of two response formats, using numbers and words. In this study, the numerical risk ratios endorsed to denote personal risk spanned the whole range of response options offered, with modal values of 1 in 10 and 1 in 50. The majority of women put their own risk at least at 2–3 times the risk they endorsed for the general population. This suggests they were using concepts of relative risk to make their ratings. The 15 women who set their own risk lower than the risk they assigned for the general population may not have understood the meaning of the ratios. These were less well-educated women, two-thirds of whom had had no formal education after the age of 16 years. Most women described themselves as 'moderately susceptible' to ovarian cancer. The remainder were equally divided between rating themselves 'very' and 'not very' susceptible. Their use of these categories was significantly related to the ratio that they endorsed to denote personal risk. The means by which risk information was communicated to the women is not the subject of this study and we made no assumptions about the terms used in the consultation. However awareness of individuals' prior estimates of their own risk is likely to be helpful in appropriately tailoring the consultation to meet individual needs.

Risk management at the clinic is operationally based on 3 risk categories i.e. high, moderate and low, derived from pedigree analysis and the woman's age at the time of the consultation. We felt it was potentially useful to identify whether or not women present with a roughly realistic perception of their own situation. We therefore used these categories as simplistic means of identifying women with under- or over-estimated risk perceptions. These women were not characterized by exaggerated perceptions of their cancer risk. Like those attending the familial breast clinic (Cull et al, 1999) they were more likely to underestimate their risk. Women with HBOC were more likely to underestimate their risk of ovarian cancer than HOC women. Among those HBOC women referred from the familial breast cancer clinic, 73% underestimated their risk of ovarian cancer. To be referred they would have had to have had at least one family member affected by ovarian cancer. Where there was a strong family history of breast cancer (e.g. 3 or 4 affected relatives) ovarian cancer may have affected only a distant relative. Typically these women had been unaware of the presence, or significance, of a family history of ovarian cancer until their history was reviewed at the familial breast clinic. Nonetheless where there was a high probability of a BRCA1/2 mutation in the family the woman's risk of ovarian cancer would be relatively high. These data suggest a particular need to monitor the impact of counselling on HBOC women who may be at greater risk of becoming distressed with increased awareness of their dual risk.

There were 34 women who were not judged by the geneticist to be at sufficiently increased risk to warrant surveillance. They should be discharged from the clinic. The available data (Table 2) show the majority of them feel 'moderately susceptible' and two of them feel 'very susceptible' to ovarian cancer. For the sample as a whole the best predictor of overestimated risk was a 'case-level' GHQ score. The danger is that the health care behaviour of these women will be driven by distress rather than objective risk. The challenge is to counsel them in such a way as to moderate their perception of their personal risk and reduce their distress while encouraging appropriate health care vigilance. The outcome of the clinic in terms of the health

**Table 5** Logistic regression to predict case-level GHQ Scores (>5)

| | Coefficient | S.E. | p value | (df) | Odds ratio | (95% confidence intervals) |
|---|---|---|---|---|---|---|
| Trait Anxiety | 0.15 | 0.03 | 0.000 | 1 | 1.16 | (1.09 – 1.23) |
| University Education | 1.70 | 0.50 | 0.001 | 1 | 5.50 | (2.08 – 14.54) |
| Estimators | | | 0.03 | 2 | | |
| Underestimators | –0.24 | 0.47 | 0.60 | 1 | 0.78 | (0.07 – 0.70) |
| Overestimators | 1.27 | 0.58 | 0.03 | 1 | 3.55 | (0.09 – 0.89) |
| Constant | –7.59 | 1.45 | 0.000 | 1 | 0.00 | |

 *© 2001 Cancer Research Campaign*

care behaviour of the distressed overestimators warrants further study. Overall, the variables included in this study explained little of the observed variation in our categorization of accuracy of women's pre-counselling risk perceptions. The assessment method used in this study was crude but this is an important construct which warrants further exploration. In Wardle's study (1995) optimism and the number of cancer deaths affecting family and friends predicted risk estimates. We have reported (Rees et al, in press) theoretical grounds for believing that a number of dimensions of personal experience of cancer may be important influences on personal risk perception.

In common with findings in other familial cancer clinics (Lloyd et al, 1996; Cull et al, 1999), most women presenting to this clinic were not highly distressed. Their GHQ scores were comparable to data from a large (UK) general population sample (Cox et al, 1987). Our data may reflect a participation bias if, as we suspect, highly distressed women avoid attending the clinic. A significant minority of our sample did return GHQ scores which warranted at least a clinical assessment of their mental health status. Some of them may require bereavement counselling for unresolved grief over family losses resulting from cancer. Women with 'case-level' distress were more than twice as likely to overestimate their risk. Other aspects of personal experience of cancer in the family e.g. recent diagnosis, close identification with the affected relative may also be salient in increasing our respondents' sense of their own susceptibility. High levels of distress for whatever reason obviate against women trying to absorb complex information about threat to their health or making informed decisions about risk management. There is therefore a need for cancer genetics services to be able to recognize clinically significant distress and to have access to appropriate referral services for those clients.

Anxiety proneness was significantly higher in our sample than in Knight et al's (1983) general population. The psychological characteristics (trait anxiety, locus of control and monitoring/blunting) observed in this sample were very similar to those observed in the familial breast clinic sample (Cull et al, 1999). Our data suggest that well educated, anxiety-prone women are more likely to present with high levels of distress about their cancer risk which they tend to overestimate. Locus of control beliefs were significantly related to distress on univariate, though not on multivariate analysis. Women who felt that their health was outwith their control were more likely to be clinically significantly distressed. This finding may be useful in planning remedial intervention. Counselling about risk management strategies might be expected to relieve these women. However we have found that the majority of women attend this clinic with exaggerated expectations of the benefits of screening (Sheppard et al, in press). Learning of the unproven efficacy of available screening methods may drive them to seek prophylactic surgery to regain control over their health and to relieve their distress. We have been exploring the factors influencing the uptake and outcome of prophylactic oophorectomy among at risk women (Fry et al, in press) but further prospective research is needed. There may also be a place for psycho-educational interventions of the kind being offered to women at increased risk of breast cancer (Kash et al, 2000). Brief group interventions which offer information and social support and which promote active coping strategies may be a cost-effective way of helping women to come to terms with familial ovarian cancer.

### ACKNOWLEDGEMENTS

We would like to acknowledge the support of the staff of the Familial Ovarian Cancer Clinic in Edinburgh, particularly the gynaecologists Dr GE Smart and Dr C Busby-Earle. Several research assistants helped with the collection of data: Hayley Miller, Susie Howat, Tracy Williamson and Amanda Barrie. The work was funded by the Imperial Cancer Research Fund.

### REFERENCES

Audrain J, Schwartz MD, Lerman C et al (1997) Psychological distress among women seeking genetic counselling for breast–ovarian cancer risk: the contribution of personality and appraisal. *Ann Behav Med* **19**(4): 370–377

Cox B, Blaxter M, Buckle A et al (1987) *The Health and Lifestyle Survey*. Cambridge: Health Promotion Research Trust

Cull A, Anderson EDC, Campbell S, Mackay J, Smyth E and Steel M (1999) The impact of genetic counselling about breast cancer risk on women's risk perceptions and levels of distress. *Br J Cancer* **79**(3/4): 501–508

Cuzick J (1985) A Wilcoxon type test for trend. *Stat Med* **4**: 87–90

Daly MB and Lerman C (1993) Ovarian cancer risk counselling: a guide for the practitioner. *Oncology* **7**(11): 27–34

Evans DGR, Burnell LD, Hopwood P and Howell A (1993) Perception of risk in women with a family history of breast cancer. *Br J Cancer* **67**: 612–614

Evans DGR, Blair V, Greenhalgh R, Hopwood P and Howell A (1994) The impact of genetic counselling on risk perception in women with a family history of breast cancer. *Br J Cancer* **70**: 934–938

Ford D, Easton DF, Peto J (1995) Estimates of the gene frequency of BRCA1 and its contribution to breast and ovarian cancer incidence. *AM J Human Genetics* **57**: 1457–1462

Fry A, Busby-Earle C, Rush R and Cull A. Long term psychosocial adjustment to prophylactic oophorectomy in women at increased risk of ovarian cancer. *PsychoOncology* (In press)

Goldberg DO and Williams P (1988) GHQ: *A Users Guide to the General Health Questionnaire*. NFER-Nelson: Windsor

Green J, Murton F, Stratham H (1993) Psychosocial issues raised by a familial ovarian cancer register. *J Med Genet* **30**: 575–579

Hallowell N and Richards MPM (1997) Understanding life's lottery: an evaluation of studies of genetic risk awareness. *J Health Psychol* **2**: 31–43

Jacobs I and Lancaster J (1996) The molecular genetics of sporadic and familial epithelial ovarian cancer. *Int J Gynaecol Cancer* **6**: 337–355

Jacobs I, Mackay J, Skates S for the UKCCCR Gynaecological Subcommittee (1997) UKCCCR National Familial Ovarian Cancer Screening study. OCS Study Registration Centre, Addenbrooke's Hospital, Cambridge

Kash KM, Dabney MK, Ortega-Vardejo et al (2000) Group intervention for women at genetic risk for breast cancer. Oral presentation to 6th International Meeting on Psychosocial Aspects of Genetic Testing for Hereditary Breast/Ovarian Cancer. Marseille, March 9th, 2000

Knight RG, Waal Manning HJ and Spears GF (1983) Some norms and reliability data for the State Trait Anxiety Inventory and Zung Self Rating Depression Scale. *Br J Clin Psychol* **22**: 245–249

Lloyd S, Watson M and Waites B (1996) Familial breast cancer: a controlled study of risk perception psychological morbidity and health beliefs in women attending for genetic counselling. *Br J Cancer* **74**: 482–487

Mackay J, Crosbie AEC, Steel CM, Smart GE and Smyth JF (1995) Clinical and ethical dilemmas in familial ovarian cancer. Chapter 8 In: Sharp F, Blackett A, Leahe R, Berek J (eds) *Ovarian Cancer*, London: Chapman-Hall

Marks G, Richardson JL, Graham JW and Levine A (1986) Role of health locus of control beliefs and expectations of treatment in adjustment to cancer. *J Pers Soc Psychol* **51**: 443–450

Miller SM (1987) Monitoring and blunting: validation of a questionnaire to assess styles of information seeking under threat. *J Pers & Soc Psychol* **52**: 345–353

Rees G, Fry A and Cull A. A family history of breast cancer. Women's experiences from a theoretical perspective. *Soc Sci & Med* (in press)

Robinson GE, Rosen BP, Bradley LN, Rockfert WG, Carr ML, Cole DEC and Murphy KJ (1997) Psychological impact of screening for familial ovarian cancer: Reactions to initial assessment. *Gynaecol Oncol* **65**: 197–205

Schwartz MD, Lerman C, Miller SM, Daly M and Maisny A (1995) Coping predisposition, perceived risk and psychological distress among women at increased risk for ovarian cancer. *Health Psychology* **14**(3) 232–235

Sheppard R, Fry A, Rush R, Steel CM and Cull A. Women at risk of ovarian cancer: attitudes towards and expectations of the familial ovarian cancer clinic. *Familial Cancer* (in press).

Spielberger C (1983) *Manual for the State Trait Anxiety Inventory*. Consulting Psychologists Press: Palo Alto, Ca

Steptoe A (1989) An abbreviated version of the Miller Behavioural Style Scale. *Br J Clin Psychol* **28**: 183–184

Stratton JF, Gayther SA, Russell P, Dearden J et al (1997). Contribution of BRCA1 mutations to ovarian cancer. *N Eng J Med* **336**: 1125–1130

Wallston KA and Wallston BS (1978). Development of the Multidimensional Health Locus of Control Scales. *Health Educ Monogr* **6**: 2 160–170

Wardle J (1995) Women at risk of ovarian cancer. *J Natl Cancer Inst Monogr No* **17**: 81–85

© 2001 Cancer Research Campaign 
